# Yet another hump for CAML: support of cell survival independent of tail-anchored protein insertion

**DOI:** 10.1038/cddis.2017.334

**Published:** 2017-07-27

**Authors:** Jennifer C Shing, Richard J Bram

**Affiliations:** 1 Department of Pediatric and Adolescent Medicine, Mayo Clinic College of Medicine and Science, Rochester, MN, USA; 2 Department of Immunology, Mayo Clinic College of Medicine and Science, Rochester, MN, USA

The endoplasmic reticulum (ER) is a structurally complex organelle that plays a central role in cell physiology. Comprised of membrane sheets and tubules, it has numerous functions, including synthesizing lipids and membranes, regulating ion homeostasis, facilitating communication between organelles, and mediating protein biogenesis.^1^ The integral ER membrane protein calcium-modulating cyclophilin ligand (CAML) was discovered in a screen for cyclophilin B-binding proteins,^2^ and has been implicated in various ER functions. For example, CAML overexpression induced constitutive calcium influx in T cells due to release of ER calcium stores.^2^ Furthermore, epithelioid *Caml*-knockout cells displayed impaired epidermal growth factor receptor recycling, establishing a role for CAML in vesicular trafficking.^3^

CAML is highly conserved in vertebrates and the protein is ubiquitously expressed.^2^ Unsurprisingly, total knockout of *Caml* is embryonically lethal,^2^ and CAML protein expression is required for survival of certain specialized immune cells.^4, 5, 6^ Thymocytes have a strict requirement for CAML during the early stages of differentiation. Mature B and T lymphocytes require CAML for proliferation and survival following antigenic stimulation, although resting lymphocytes are viable without the protein. Furthermore, mouse embryonic fibroblasts (MEFs), acutely depleted of CAML did not undergo apoptosis, but developed multiple mitotic defects and aneuploidy,^7^ while mouse embryonic stem cells from *Caml*-knockout embryos grow readily.^3^ Thus, CAML is not a constitutive survival factor for all cells, but must play a specific role in particular cell types.

Recent findings identified a role for CAML in facilitating tail-anchored (TA) protein insertion. TA proteins comprise a unique subset of integral membrane proteins bearing a single, hydrophobic transmembrane domain (TMD) close to the C-terminus.^8, 9^ This TMD serves as a membrane anchor, allowing TA proteins to be properly localized to membrane-bound organelles. During TA protein biogenesis, the ribosomal exit tunnel prevents the C-terminal TMD from interacting with co-translational insertion machinery; therefore, translocation of TA proteins into the ER membrane occurs in a post-translational manner. TA proteins comprise ~3 to 5% of all integral membrane proteins,^9^ and include proteins found in all membranes, such as Bak, Sec61*β* and Pex26.^8^ Individual TA proteins vary in their requirement for TA insertion machinery in order to attain their proper membrane insertion.^8, 10^

In mammals, the cytosolic TMD recognition complex of 40 kDa (TRC40) ATPase is responsible for delivering TA proteins to the ER membrane for translocation.^11^ In the TRC40 pathway, TRC40 interacts with the TA protein client, which it receives from a pre-targeting complex. TA proteins are then transported to an ER-resident receptor, which directs its insertion into the membrane. CAML was discovered to associate with TRC40,^12^ and was found to serve as the mammalian receptor for TRC40 in association with tryptophan-rich basic protein (WRB).

Given the multiplicity of TA proteins that may depend on CAML for proper subcellular localization, it has been postulated that this mechanism may underlie the many pleiotropic functions of CAML previously identified, including cell survival, mitosis, calcium signaling and membrane trafficking.^13^ In our new study published in *Cell Death Discovery*,^14^ we present evidence indicating that CAML is required for the survival of Myc-driven lymphomas and demonstrate that the pro-survival function of CAML cannot be explained by its role in TA protein insertion. For this study, we generated *Eμ-Myc* transgenic mice carrying a tamoxifen-inducible conditional knockout allele of *Caml*. B-cell lymphoma cell lines developed from these mice were used to determine whether CAML is required for lymphoma growth.

*Eμ-Myc* lymphoma cells died by apoptosis following homozygous *Caml* deletion induced by 4-hydroxytamoxifen treatment, indicating that CAML is required for the survival of Myc-driven B-cell lymphomas. Tumor growth in mice also required CAML expression because adoptively engrafted lymphomas derived from a similar cell line regressed after tamoxifen administration. Interestingly, suppression of apoptosis by overexpression of Bcl-2 or Bcl-x_L_ did not restore proliferation of *Caml*-deleted cells. This observation indicated that CAML supports cell survival and proliferation independently. We therefore investigated the cell cycle of *Caml*-deleted cells and found an arrest in G2/M phase, which was likely due to delayed exit from M phase. We conclude that CAML has a role in mitotic progression, consistent with previous findings in MEFs.^7^

To identify the domains of CAML required for cell survival and growth, we performed structure-function analyses by expressing CAML deletion mutants in knockout cells. Using this approach, we determined that the N-terminal domain of CAML, which allows interaction of CAML with TRC40, was dispensable for the survival of *Caml*-deleted *Eμ-Myc* cells. Moreover, a minimal C-terminal transmembrane region of CAML was able to rescue *Caml*-deleted *Eμ-Myc* cells. Because the C-terminal transmembrane portion of CAML associates with WRB in the ER membrane,^12^ we wondered if the CAML C-terminus could still support TA protein translocation. Consistent with the structure-function analysis of the N-terminus, the CAML C-terminus was unable to restore TRC40-dependent TA protein insertion in *Caml*-deleted cells, verifying that TA protein insertion is not required for the survival and proliferation of *Caml*-deleted cells. Instead, we conclude that a TA-independent role accounts for the pro-survival function of CAML (Figure 1).

This study is the first to examine CAML function in a cancer model, and shows that CAML is required for cell viability and proliferation of a rapidly growing type of B-cell lymphoma. These results are consistent with previous findings in activated immune cells, which demonstrated that CAML is required for survival post-stimulation but not in resting lymphocytes.^4, 5–6^ Additionally, the cell cycle analysis of *Caml*-deleted *Eμ-Myc* cells indicated that CAML is required for mitotic progression. This data aligns with earlier observations in *Caml*-knockout MEFs showing weakened spindle assembly checkpoint and mitotic segregation errors,^7^ and suggests that this defect may also explain the lethality of *Eμ-Myc* cells lacking CAML.

While CAML has been found to be involved in many processes including calcium signaling, receptor recycling and the spindle assembly checkpoint,^2, 3, 7, 13^ it is unknown whether all of these functions are exerted independently of TA protein translocation, or whether these roles can partially be explained by TA protein biogenesis. The observation that endogenous CAML is present in five-fold molar excess over endogenous WRB raises the question about why excess CAML protein is present in cells.^15^ Because the C-terminal region of CAML maintains the ability to interact with WRB, CAML and WRB could function together in a non-TA protein insertion role. Alternatively, a WRB-free pool of CAML, which can interact with other protein partners could support cell viability.

Taken together, the evidence we have presented strengthens the idea that CAML possesses pro-survival function in addition to its known role in TA protein insertion. Future investigations should delve further into the mechanisms by which CAML mediates cell survival, and explore how its pleiotropic roles relate to membrane protein translocation. Continued discoveries about this multifaceted protein may reveal novel aspects of ER function in cell physiology, and the detailed mechanism that underlies mitotic segregation and post-translational processing of membrane proteins.

## Figures and Tables

**Figure 1:**
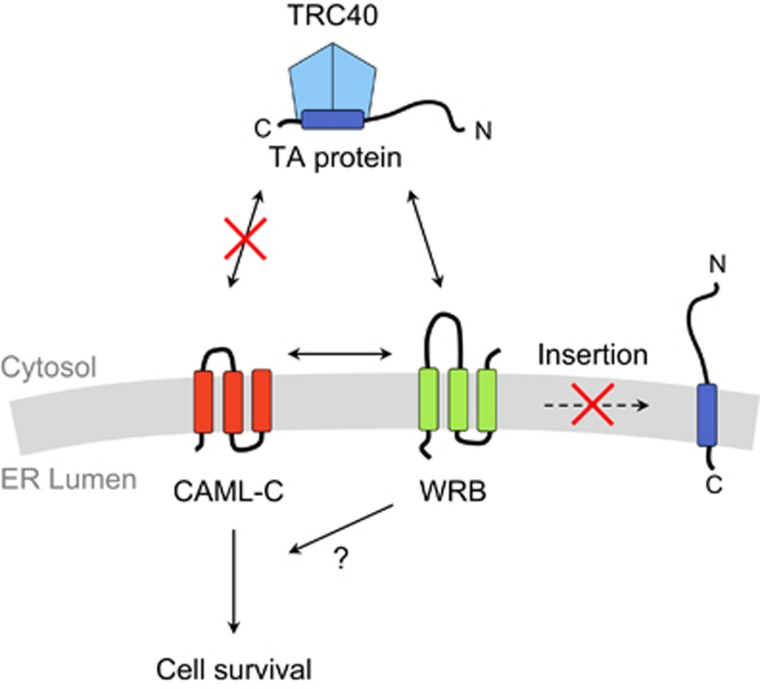
The C-terminal region of CAML interacts with WRB and facilitates cell survival, but not TA protein insertion. By expressing the C-terminal 111 amino acid residues of human CAML (CAML-C), the C-terminal region was determined to bind to WRB but not TRC40. The C-terminal fragment of CAML did not facilitate TA protein insertion, but supported cell survival and proliferation of *Caml*-deleted *Eμ-Myc* cells. It is unknown whether CAML and WRB cooperate to promote cell viability, or if CAML alone or in combination with other partners supports survival
